# Flavor’s Delight

**DOI:** 10.3390/e26050355

**Published:** 2024-04-24

**Authors:** Hans Peter Nilles, Saúl Ramos-Sánchez

**Affiliations:** 1 Bethe Center for Theoretical Physics, Physikalisches Institut der Universität Bonn, Nussallee 12, 53115 Bonn, Germany; nilles@th.physik.uni-bonn.de; 2 Instituto de Física, Universidad Nacional Autónoma de México, Ciudad de México C.P. 04510, Mexico

**Keywords:** flavor, string compactifications, eclectic symmetries

## Abstract

Discrete flavor symmetries provide a promising approach to understand the flavor sector of the standard model of particle physics. Top-down (TD) explanations from string theory reveal two different types of such flavor symmetries: traditional and modular flavor symmetries that combine to the eclectic flavor group. There have been many bottom-up (BU) constructions to fit experimental data within this scheme. We compare TD and BU constructions to identify the most promising groups and try to give a unified description. Although there is some progress in joining BU and TD approaches, we point out some gaps that have to be closed with future model building.

## 1. Introduction

The problem of flavor, the description of masses and mixing angles of quarks and leptons, remains one of the most important questions in elementary particle physics. A major approach to solve this problem is based on non-Abelian (discrete) flavor symmetries. In attempting to fit presently available data, many different symmetries and representations of flavor groups have been suggested and analysed. A comprehensive summary of these BU attempts can be found in the reviews [1,2,3,4]. In his book [5] with Jihn E. Kim, entitled *History of Particle Theory*, Paul Frampton (p. 172) mentions his preferred flavor group $T^{\prime }$, the binary tetrahedral group. This choice is motivated through his early work on flavor symmetries: see ref. [6] and references therein.

Most attempts in the BU approach focus on the lepton sector to obtain solutions close to neutrino tribimaximal mixing [7]. Prominent examples have been $A_{4}$, $S_{4}$, Δ(27), Δ(54), Σ(81), and Q(24), among many others [8]. While they lead to acceptable solutions in the lepton sector, applications to the quark sector have been less frequent and usually less successful. Still, as there are many viable models it is difficult to draw a definite conclusion about the correct choice.

It seems that we need additional ingredients to select models from a more theoretical point of view. Such TD considerations draw their motivation from string theory model building, in particular orbifold compactifications of the heterotic string [9,10,11,12,13,14]. Early work [15] on the $Z_{3}$ orbifold revealed the discrete flavor group Δ(54) with irreducible triplet representations to describe the three families of quarks and leptons. Even more earlier work, analyzing duality symmetries in string theory [16,17,18,19], provided an example of the discrete (modular) group $T^{\prime }$. From this point of view, the predictions of the $Z_{3}$ orbifold lead to the discrete groups Δ(54) and $T^{\prime }$.

Fortunately, these groups allow many connections to models of the BU approach; in fact, $T^{\prime }$ and Δ(54), as well as their “little sisters” $A_{4}$ and Δ(27), have played a major role (for an encyclopedia of discrete groups and technical details, we refer to ref. [20]). In the following, we want to analyze these specific constructions in detail. In Section 2, we start with the tetrahedral group *T* (isomorphic to the group $A_{4}$ of even permutations of four objects), which played a major role in the discussion of neutrino tribimaximal mixing. We continue with its double cover $T^{\prime }$ and potential applications to flavor physics. Section 3 introduces the motivation for the use of the group Δ(27) for leptonic mixing. It has 27 elements and is a discrete subgroup of SU(3). It is also a subgroup of Δ(54) that appeared in early discussions of flavor groups in string theory constructions [15]. Section 4 is devoted to TD considerations of flavor symmetries from string theory model building. There, we shall also introduce the concept of discrete modular symmetries that were discovered from an analysis of dualities in string theory [16,17,18,19]. The application of modular symmetries to flavor physics was pioneered in the BU approach by Feruglio [21] for the example of the discrete modular group $A_{4}$. We argue that the TD approach favors instead the modular flavor group $T^{\prime }$, the double cover of $A_{4}$. Section 5 introduces the concept of the eclectic flavor group [22,23] that appears as a prediction in the string theory framework. It combines the traditional flavor symmetries (here, Δ(54)) with the discrete modular flavor symmetries (here, $T^{\prime }$). In Section 6, we shall try to make contact between the BU and TD approaches. Section 7 will give an outlook on strategies for further model building. The appendices will provide technical details of the properties of $A_{4}$, $T^{\prime }$, Δ(27), and Δ(54).

## 2. The Tetrahedral Group and Its Double Cover

The symmetry group *T* of the tetrahedron is one of the smallest non-Abelian discrete groups and found early applications in particle physics [24,25]. It has 12 elements and is isomorphic to $A_{4}$, the group of even permutations of four elements. There are three singlets $\left(1,1^{\prime },1^{\prime \prime }\right)$ and one irreducible triplet representation. Detailed properties of $T≅A_{4}$ can be found in Appendix A.1. The presence of the triplet representation makes it attractive for flavor physics with three families of quarks and leptons. It became particularly relevant for the discussion of (nearly) tribimaximal mixing [26,27] in the lepton sector. An explicit discussion of this situation can be found in the reviews [1,8]. Tribimaximal mixing [7] is characterized (up to phases) through the PMNS structure

$$U_{\mathrm{PMNS}}\;=\;\left(\begin{array}{ccc} \sqrt{\frac{2}{3}} & \frac{1}{\sqrt{3}} & 0\\ -\frac{1}{\sqrt{6}} & \frac{1}{\sqrt{3}} & -\frac{1}{\sqrt{2}}\\ -\frac{1}{\sqrt{6}} & \frac{1}{\sqrt{3}} & \frac{1}{\sqrt{2}} \end{array}\right)$$

and includes a $Z_{2}$ × $Z_{2}$ symmetry acting (in the neutrino mass basis) as U=diag(−1,−1,1) and V=diag(−1,1,−1). This symmetry is a subgroup of $S_{4}$, the group of permutations of four elements. Tribimaximal mixing, however, is not exactly realized in nature as it would imply that the (reactor) angle $\theta_{13}$ vanishes. The $Z_{2}$ transformation *V* thus cannot be an exact symmetry. This brings $A_{4}$ into the game, a subgroup of $S_{4}$ that does not contain *V*. It allows satisfactory fits for the lepton sector, as reviewed in ref. [1]. These applications typically use the triplet representation for the left-handed lepton-SU(2)-doublets $\left(\nu_{i},\ell_{i}\right)$ and the representations $(1,1^{\prime }$ and $1^{\prime \prime })$ for the the SU(2) singlets of the standard model of particle physics (SM). Various “flavon” fields have to be considered for the spontaneous breakdown of $A_{4}$, and this is subject to explicit model building, which we shall not discuss here in detail. In any case, $T≅A_{4}$ is a very appealing discrete flavor symmetry for the description of the lepton sector.

A look at the quark sector reveals a completely different picture: there, all mixing angles are small and a fit similar to the lepton sector does not seem to work. One particular property of the quark sector is the fact that the top-quark is much heavier than the other quarks. This seems to indicate a special role of the third family, somewhat sequestered from the other two families. This could therefore imply that for quarks the third-family is a singlet under the discrete flavor group. Such a situation can be well described in the framework of $T^{\prime }$, the double cover of $T≅A_{4}$. This group has 24 elements with representations $1,1^{\prime },1^{\prime \prime },3$ (as $A_{4}≅T$) and in addition doublet representations $2,2^{\prime },2^{\prime \prime }$ (details of properties of $T^{\prime }$ can be found in Appendix A.2). This double-cover is similar to the double-cover SU(2) of SO(3) when describing angular momentum. In fact, *T* is a subgroup of SO(3) and $T^{\prime }$ is a subgroup of SU(2). This implies that the dynamics and constraints associated with *T* can equally stem from the larger group $T^{\prime }$ (in analogy to the fact that one can also describe integer spin with SU(2)), while the doublet representations of $T^{\prime }$ allow for more options [28,29].

This fact has been used in refs. [30,31] to obtain a simultaneous description of both, the lepton- and the quark-sector in the framework of $T^{\prime }$ [6]. The lepton sector remains the same as in the $A_{4}$ case, while in the quark sector we do not use the irreducible triplet representation, but the representation $1⊕2^{\prime }$, to single out the third family. This seems to be a nice explanation of the difference of the quark and lepton sectors within the flavor group $T^{\prime }$. As Paul Frampton says in his book with Jihn E. Kim [5] (page 172) “Clearly, it is better simultaneously to fit both the quark- and lepton-mixing matrices. This is possible using, for example, the binary tetrahedral group $T^{\prime }$”. There are, of course, many other attempts based on larger groups and representations, but $T^{\prime }$ remains a very attractive option.

## 3. Towards Larger Groups: Δ(27) and Δ(54)

Although small groups such as $A_{4}$ and $T^{\prime }$ already lead to satisfactory fits, there are a lot of new parameters and ambiguities in explicit model building, and it is not evident whether this really gives the ultimate answer. In fact, there have been many more attempts with different groups and different representations, as can be seen in refs. [1,2,3,4]. Another attractive small group is Δ(27). It has 27 elements and 9 one-dimensional representations, as well as a triplet 3 and an anti-triplet $\bar{3}$ representation. Technical details of the group are given in Appendix B.2. This is still a small group and is attractive in particular because of the 3 and $\bar{3}$ representations, which are well suited for flavor model building with three families of quarks and leptons. As shown in the appendix, it can be constructed as a semi-direct product of $Z_{3}$ × $Z_{3}$ and $Z_{3}$ and is a subgroup of SU(3).

Early applications can be found in refs. [32,33,34,35,36], which exploit the presence of the 3 and $\bar{3}$ representations. For more recent work and a detailed list of references, refer to refs. [37,38]. As in the case of $A_{4}$, Δ(27) is also well suited to accommodate near tribimaximal mixing. Again (as for $A_{4}$), the $Z_{2}$ × $Z_{2}$ group of tribimaximal mixing is not a subgroup of Δ(27), but it appears approximately for specific alignments of the vacuum expectation values of flavon fields that appear naturally within Δ(27).

Δ(27) is the “little sister” and subgroup of Δ(54). This group has 54 elements, two singlet, four doublet, and two pairs of triplet and anti-triplet ($3⊕\bar{3}$) representations. The properties of Δ(54) are collected in Appendix B.1. It is already quite a large group, somewhat unfamiliar to the BU flavor-community and found less applications than Δ(27). It became popular because of its appearance in string theory [15], which we shall discuss in Section 4 in detail. Explicit BU model building with Δ(54) was pioneered in ref. [20].

## 4. Top-Down Considerations: A Taste of Flavor from String Theory

In the BU approach, there are many successful models based on various groups and representations [1,2,3,4], too many to single out a “best” option. Such an answer might come from theoretical considerations and top-down model building. An attractive framework is provided by string theory. Here we shall concentrate on the orbifold compactifications of heterotic string theory, which provide many realistic models with gauge group SU(3) × SU(2) × U(1) and three families of quarks and leptons [10,13,39,40,41,42,43,44].

In these theories, discrete flavor symmetries arise as a result of the geometry of extra dimensions and the geography of fields localized in compact space. Strings are extended objects, and this reflects itself in generalized aspects of geometry that include the winding modes of strings. A full classification of the flavor symmetries of orbifold compactifications of the heterotic string is given by the outer automorphisms [45,46] of the Narain space group [47,48,49,50]. Here we shall not be able to give a full derivation of this fact, but only provide a glimpse of the general TD formalism and illustrate the results in simple examples based on a D=2-dimensional torus and its orbifold.

In general, a string in *D* dimensions has *D* right-moving and *D* left-moving degrees of freedom, encoded in $Y=(y_{R},y_{L})$. Compactifying the theory on a *D*-dimensional torus demands that the 2D degrees of freedom be subject to the toroidal boundary conditions

$$Y\;=\;\left(\begin{array}{c} y_{R}\\ y_{L} \end{array}\right)\;∼\;Y+E\;\hat{N}\;=\;\left(\begin{array}{c} y_{R}\\ y_{L} \end{array}\right)+E\left(\begin{array}{c} n\\ m \end{array}\right),$$

where the winding and the Kaluza–Klein (momentum) quantum numbers of the string, $n,m\in Z^{D}$, define a 2D-dimensional Narain lattice. *E* denotes the so-called Narain vielbein and contains the moduli $M_{i}$ of the torus. In the Narain formulation, we achieve a *D*-dimensional orbifold by imposing the identifications

$$Y∼\Theta^{k}\;Y+E\;\hat{N},\;\mathrm{with}\;\mathrm{the}\;Z_{K}\;\mathrm{orbifold}\;\mathrm{twist}\;\Theta =\left(\begin{array}{cc} \theta_{R} & 0\\ 0 & \theta_{L} \end{array}\right)\;\mathrm{satisfying}\;\Theta^{K}=1_{2D}\;,$$

where k=0,…,K−1 and the SO(D) elements $\theta_{L},\theta_{R}$ are set to be equal to obtain a symmetric orbifold. Excluding roto-translations, the Narain space group can then be generated by

$$\mathrm{the}\;\mathrm{twist}\;\;\left(\Theta ,0\right)\;\;\mathrm{and}\;\mathrm{shifts}\;\;\left(1,E_{i}\right)\;\;\mathrm{for}\;\;i=1,\ldots ,2D\;.$$

It turns out that flavor symmetries correspond to the (rotational and translational) outer automorphisms of this Narain space group [45,46], which are transformations that map the group to itself but do not belong to the group.

Hence, it follows that the flavor symmetries of string theory come in two classes:

- Those symmetries that map momentum- to momentum- and winding- to winding-modes. These symmetries we call traditional flavor symmetries. They are the same type as those symmetries that would appear in a quantum field theory of point particles. In the Narain formulation, these can be understood as translational outer automorphisms of the Narain space group.
- Symmetries that exchange winding- and momentum-modes. They have their origin in the duality transformations of string theory. We call them modular flavor symmetries as (for the torus discussed here) they are connected to the modular group SL(2,Z). These arise from rotational outer automorphisms of the Narain space group.

### 4.1. Traditional Flavor Symmetries

Here we concentrate on the two-dimensional cases $T^{2}/Z_{K}$ and K=2,3,4,6, which can be understood as the fundamental building blocks for the discussion of flavor symmetries. They have been discussed in detail in ref. [15]. Various groups can be obtained, prominently $D_{8}$ or Δ(54). As an illustrative example, we discuss here the case $T^{2}/Z_{3}$ with group Δ(54) because it has the nice property to provide irreducible triplet representations for three families of quarks and leptons [43,51].

**Figure 1:**
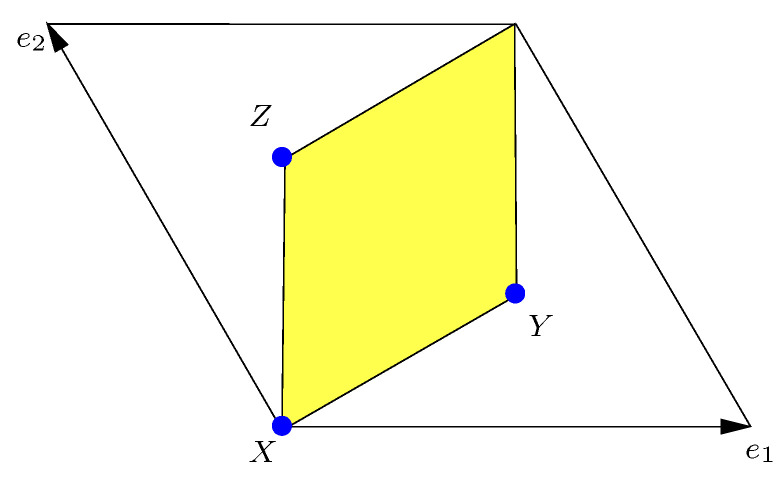
The $T^{2}/Z_{3}$ orbifold (yellow shaded region) with three fixed points, X,Y,Z. Twisted states are localized at theses fixed points. Figure taken from ref. [54].

The $Z_{3}$ orbifold $T^{2}/Z_{3}$ is shown in Figure 1. Twisted fields are localized on the fixed points X,Y,Z of the orbifold. This geometry leads to an $S_{3}$ symmetry from the interchange of the fixed points. String theory selection rules provide an additional $Z_{3}$ × $Z_{3}$ flavor symmetry, as discussed in ref. [15]. The full traditional flavor symmetry is Δ(54), the multiplicative closure of these groups. The twisted states on the fixed points X,Y,Z transform as (irreducible) triplets under Δ(54) (details can be found in Appendix B.1). Δ(54) has two independent triplet representations, $3_{1}$ and $3_{2}$. Both can be realized in string theory, depending on the presence or absence of twisted oscillator modes [23]. The untwisted states are in the trivial 1 representation in the absence and $1^{\prime }$ in the presence of oscillator modes. A nontrivial vacuum expectation value of a field in the $1^{\prime }$ representation will break Δ(54) to Δ(27). A discussion of the breakdown pattern of Δ(54) can be found in ref. [52]. Winding states transform as doublets under Δ(54). They are typically heavy and could play a prominent role in the discussion of CP-violation in string theory as they provide a mechanism for baryogenesis through the decay of the heavy winding modes [53].

### 4.2. Modular Flavor Symmetries

Modular flavor symmetries have their origin in the duality transformations of string theory. One example is *T*-duality, which exchanges winding and momentum modes. As a warm-up example, consider a string on a circle of radius *R*.

The masses of momentum modes are governed by 1/R, while winding states become heavier as *R* grows. On the other hand, the T-duality of string theory is defined by the transformations

$$\mathrm{winding}\;\mathrm{modes}\;⟷\;\mathrm{momentum}\;\mathrm{modes}\;\mathrm{and}\;R\;⟷\;\alpha^{\prime }/R\;.$$

Hence, T-duality maps a theory to its T-dual, which coincides at the self-dual point $R^{2}=\alpha^{\prime }=1/M_{\mathrm{string}}^{2}$, where $1/\alpha^{\prime }$ is the string tension. For a generic value of the modulus *R*, T-duality exchanges light and heavy states, which suggests that T-duality could be relevant to flavor physics. Since string theory demands the compactifications of more than one extra dimension, T-duality generalizes to large groups of nontrivial transformations of the moduli of higher-dimensional tori. For instance, in D=2 the transformations on each of the (Kähler and complex structure) moduli build the modular group $\mathrm{SL}{\left(2,Z\right)}^{2}$ of the $T^{2}$ torus. The group SL(2,Z) is generated by two elements,

$$S\;\;\mathrm{and}\;\;T,\;\mathrm{such}\;\mathrm{that}\;S^{4}\;=\;1\;,\;S^{2}T\;=\;TS^{2},\;\mathrm{and}\;{\left(ST\right)}^{3}\;=\;1\;.$$

For each modular group, SL(2,Z), there exists an associated modulus, *M*, that transforms as

$$S:\;M\;\mapsto \;-\frac{1}{M}\;\mathrm{and}\;T:\;M\;\mapsto \;M+1\;.$$

**Figure 2:**
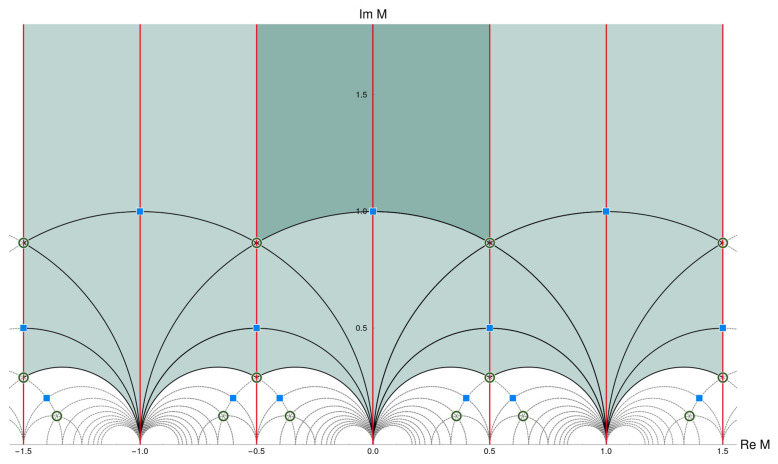
Fundamental domain of SL(2,Z) (dark shaded) and of its subgroup Γ(3)≅SL(2,3Z) (light shaded). Figure taken from ref. [54].

Further transformations include mirror symmetry (which exchanges Kähler and complex structure moduli) as well as the CP-like transformation

$$U:\;M\;\mapsto \;-\bar{M}\;,$$

where $\bar{M}$ denotes the complex conjugate of *M*. String dualities give important constraints on the action of the theory via the modular group SL(2,Z) (or GL(2,Z) when including U). A general SL(2,Z) transformation of the modulus is given by

$$M\;\overset{\gamma }{⟼}\;\frac{a\;M+b}{c\;M+d},\;\gamma \;=\;\left(\begin{array}{cc} a & b\\ c & d \end{array}\right)\;\in \;\mathrm{SL}\left(2,Z\right),$$

with detγ=1 and a,b,c,d∈Z. The value of *M* (originally in the upper complex half plane) is then restricted to the fundamental domain, as shown in (the dark shaded region of) Figure 2. Matter fields ϕ turn out to transform as

$$\phi \;\overset{\gamma }{⟼}\;{\left(c\;M+d\right)}^{k}\rho \left(\gamma \right)\;\phi \;\mathrm{for}\;\gamma \;\in \;\mathrm{SL}\left(2,Z\right)\;,$$

where ${\left(c\;M+d\right)}^{k}$ is known as automorphy factor, *k* is a modular weight fixed by the compactification properties [55,56], and ρ(γ) is a unitary representation of γ. Interestingly, ${\left(\rho \left(T\right)\right)}^{N}=1$ even though $T^{N}\neq 1$, such that ρ(γ) generates a so-called finite modular group, as we shall shortly discuss. Among others, the modular weights, *k*, of the fields are important ingredients for flavor model building.

As in the one-dimensional case, duality maps one theory to its dual, and there remains the question of whether or not such transformations are relevant for the low-energy effective action of the massless fields. This has been discussed explicitly with the help of worldsheet conformal field theory methods [16,17,18,19]. It leads to field-dependent Yukawa couplings that transform as modular forms $Y^{\left(n_{Y}\right)}\left(M\right)$ of a weights $n_{Y}>0$,

$$Y^{\left(n_{Y}\right)}\left(M\right)\;\overset{\gamma }{⟼}\;{\left(c\;M+d\right)}^{n_{Y}}\rho_{Y}\left(\gamma \right)\;Y^{\left(n_{Y}\right)}\left(M\right)\;\mathrm{for}\;\gamma \;\in \;\mathrm{SL}\left(2,Z\right)\;,$$

where, as for matter fields, $\rho_{Y}\left(\gamma \right)$ is also a unitary representation of γ in a finite modular group. The description in terms of supergravity actions has been given in ref. [57]. From the transformation properties of matter fields and Yukawa couplings, it becomes clear that the action is subject to both invariance under the finite modular group and conditions on the modular weights, which are strongly restricted in the TD approach.

Let us illustrate the relevance to flavor physics in the case of the $Z_{3}$ orbifold. We start with a two-torus and its two moduli: Kähler modulus *M* and complex structure modulus *U*. On the orbifold, the *U*-modulus is frozen, such that the lengths of the lattice vectors $e_{1}$ and $e_{2}$ are equal, with an angle of 120 degrees (see Figure 1). This also gives restrictions on the modular transformations of the matter fields. The coefficients a,b,c,d∈Z of the modular transformation are defined only by modulo 3; hence, instead of the full modular group SL(2,Z), we have to deal with its so-called principal congruence subgroup (the principal congruence subgroup of level *N* is denoted by Γ(N) and built by all γ∈SL(2,Z), such that γ=1modN), Γ(3)≅SL(2,3Z). Clearly, Γ(3) still has infinitely many elements, but it is a normal subgroup of the finite index in SL(2,Z). Hence, a finite discrete modular group can be obtained by the quotient $\mathrm{SL}\left(2,Z\right)/\Gamma \left(3\right)=\Gamma_{3}^{\prime }$. An explicit discussion is provided in ref. [58]. $\Gamma_{3}^{\prime }$ is isomorphic to $T^{\prime }$, the binary tetrahedral group. It is the double cover of $\Gamma_{3}≅A_{4}$, which one would obtain starting with PSL(2,Z) instead of SL(2,Z). In the first application of discrete modular flavor symmetry, Feruglio [21] used the group $\Gamma_{3}≅A_{4}$ with its representations $1,1^{\prime },1^{\prime \prime }$, and 3 to explain tribimaximal mixing in the standard way. Complications with flavon fields and many additional parameters could be avoided as the modular flavor symmetry is nonlinearly realized. This might lead to problems with the control of additional free parameters in the Kähler potential, which has been taken into account [59]. The modular flavor approach was picked up quickly [2,4,60,61,62,63,64,65,66] and led to many different BU constructions with various groups, representations of modular weights.

Unfortunately, the TD approach is much more restrictive and allows less freedom in model building. In our example, we obtain $T^{\prime }$ and not $A_{4}$ (the double cover is necessary to obtain chiral fermions in the string construction). Moreover, the twisted states do not transform as irreducible triplets of $T^{\prime }$ but as $1⊕2^{\prime }$, and the modular weights of the fields are correlated with the $T^{\prime }$ representation (and thus cannot be chosen freely as done in the BU framework). Some details of $T^{\prime }$ modular forms are provided in Appendix C.

## 5. Eclectic Flavor Groups

So far we have seen that string theory predicts the presence of both the traditional flavor group (Δ(54) in our example) and the modular flavor group ($T^{\prime }$). You cannot have one of them without the other. This should be taken into account in flavor model building. The eclectic flavor group [22] is the multiplicative closure of Δ(54) and $T^{\prime }$, here Ω(1)=[648,533]. (We have somewhat simplified the discussion here. In full string theory with six compact extra dimensions, we usually find additional *R*-symmetries that would increase the eclectic flavor group, here the group Ω(1) to Ω(2)=[1944,3448]. A detailed discussion of these subtleties can be found in refs. [67,68].) Observe that this group has only 648 elements for the product of groups with 54 and 24 elements, respectively. There is one $Z_{2}$-like element contained in both Δ(54) and $T^{\prime }$. Incidentally, this is the same element that enhances Δ(27) to Δ(54). Thus, Δ(27) and Δ(54), together with $T^{\prime }$, would lead to the same eclectic group [22].

**Figure 3:**
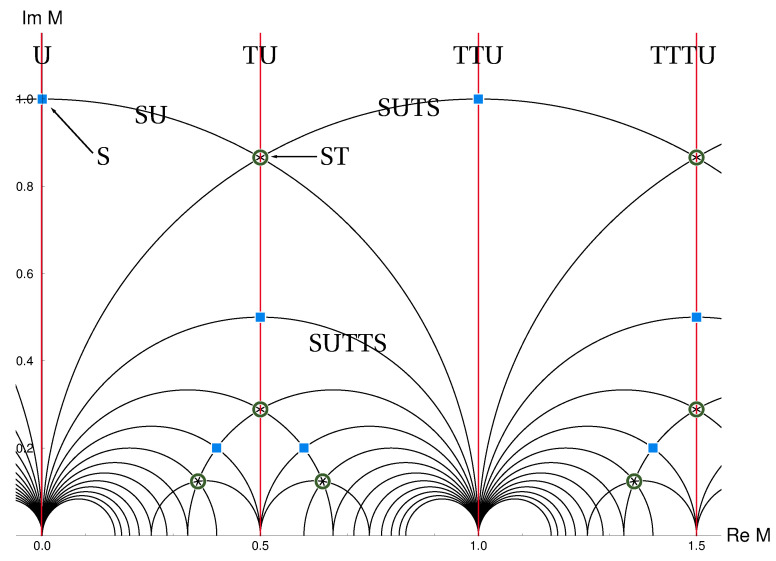
Unbroken modular symmetries at special curves in moduli space, including the CP-like generator U, which maps $M\mapsto -\bar{M}$. Figure adapted from ref. [46].

The eclectic flavor group is nonlinearly realized. Part of it appears “spontaneously” broken through the vacuum expectation value of the modulus *M*. The modulus is confined to the fundamental domain of Γ(3)=SL(2,3Z), as displayed in Figure 2. This area is reduced by a factor two if we include the natural candidate for a CP-symmetry that transforms *M* to $-\bar{M}$. The CP-symmetry extends SL(2,Z) to GL(2,Z), $T^{\prime }$ to GL(2,3) (a group with 48 elements), and the eclectic group Ω(1) to a group with 1296 elements. The fundamental domain includes fixed points and fixed lines with respect to the modular transformations S and T as well as the CP-transformation $U:M\to -\bar{M}$, as shown in Figure 3.

**Figure 4:**
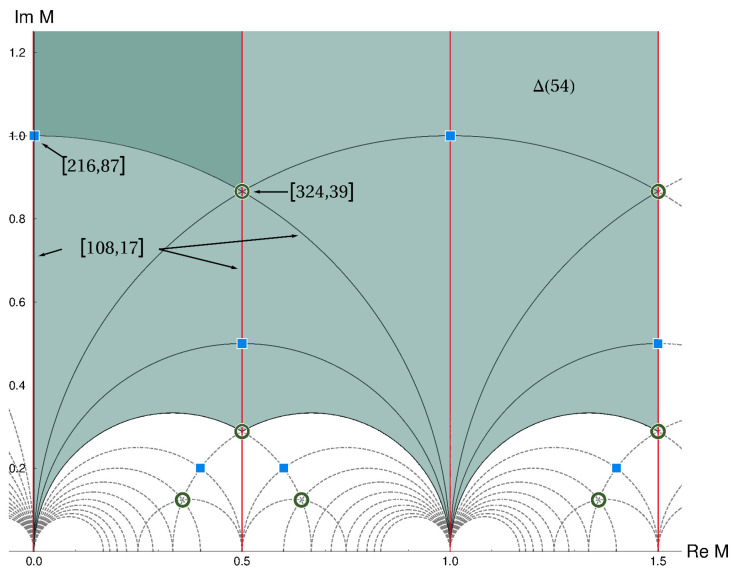
Local flavor unification at special points and curves in moduli space. The traditional flavor symmetry Δ(54), valid at generic points, is enhanced to two (different) groups with GAP Id [108,17] at the vertical lines and semi-circles, including CP-like transformations. At the intersections of curves, the flavor symmetry is further enhanced to [216,87] and [324,39], also with CP-like transformations. Figure adapted from ref. [45].

For generic points in moduli space the traditional flavor symmetry, Δ(54) is linearly realized. At the fixed points and lines this symmetry is enhanced to larger groups, as illustrated in Figure 4.

We see that here the largest linearly realized group has 324 elements with GAP Id [324,39]. (We use the group notation of the classification of GAP [69].) Thus, only part of the full eclectic flavor group with 1296 elements (including CP) can be linearly realized. The enhancement of the traditional flavor symmetry at fixed loci (here points and lines) in the fundamental domain exhibits the phenomenon called “Local Flavor Unification” [45,46]. The flavor symmetry is non-universal in moduli space, and the spontaneous breakdown of modular flavor symmetry can be understood as a motion in moduli space. This has important consequences for flavor model building. At the loci of enhanced symmetry, some of the masses and mixing angles of the quark- and lepton-sector might vanish. The explanation of small parameters and hierarchies in flavor physics can thus find an explanation if the modulus is located close to the fixed points or lines [52,70,71,72,73,74,75,76,77,78]. The mechanism of moduli stabilization in string theory could therefore provide the ingredients to understand the mysteries of flavor [71,79,80,81].

## 6. Top-Down Does Not Yet Meet Bottom-Up

There have been many BU constructions, but only a few take TD considerations into account [82,83,84]. From the presently available TD models, the groups Δ(54) for traditional and $T^{\prime }$ for modular flavor symmetry seem to be the favorite choices. In fact, there is only one explicit model that incorporates the SM with gauge group SU(3) × SU(2) × U(1) and three families of quarks and leptons [72]. We certainly need more work in the TD approach. Therefore, any conclusions about the connection between the two approaches is necessarily preliminary. Still, it is reassuring to see that the same groups Δ(54) and $T^{\prime }$ and their “little sisters” Δ(27) and $A_{4}$ appear prominently in BU constructions. One could therefore try to make contact between the two approaches within this class of models.

Before we do that, we would like to stress some important properties of the TD approach that seem to be of more general validity and thus should have an influence on BU model building. The first of these is the prediction of string theory for the simultaneous presence of both traditional and modular flavor symmetry that combine to the eclectic flavor group. It is this eclectic group that is relevant, not one of the others in isolation. Up to now, many BU constructions only consider one of them. Therefore, a direct contact between the two approaches is very difficult at this point.

The TD approach is very restrictive. Apart from the limited type of groups that appear in the TD constructions, there are also severe restrictions on the choice of representations. Not everything is possible. In the case of modular symmetry $T^{\prime }$, for example, the irreducible triplet representation does not appear in the spectrum, while many BU constructions exactly concentrate on this representation. Therefore, the TD approach cannot make contact with models based on modular $A_{4}$ flavor symmetry, where these triplets are generally used. For $T^{\prime }$, we have the twisted fields in the $1⊕2^{\prime }$ representation. It seems to be more likely that irreducible triplet representations are found within the traditional flavor group, as seen in the example with Δ(54).

A second severe restriction concerns the choice of modular weights. In the TD approach we have essentially no choice. Once we know the representations of the eclectic group, the modular weights are fixed. This is an important restriction, as in the BU approach the choice of modular weights is an important ingredient of model building. With a careful choice of modular weights one can create additional “shaping symmetries”, which are important for the success of the fit to the data. This is not possible in the TD approach. There the role of such symmetries could, however, be found in the traditional flavor symmetry.

As a result of these facts, there is presently still a crucial difference between the BU and TD approaches, and a direct comparison is not possible at this point. We are still at the very early stage of such investigations.

## 7. Outlook

Much more work in both approaches is needed in order to clarify the situation. In the BU approach, it would be desirable to consider models that fulfill the restrictions coming from TD. Traditional flavor symmetries and the eclectic framework should be taken into account. A toolkit for such a construction can be found in the consideration of a modular group that fits into the outer automorphism of the traditional flavor group, as explained in ref. [22]. A recent application of this connection for the traditional flavor group Δ(27) has been discussed in refs. [83,84]. Moreover, BU constructions should avoid the excessive use of modular weights in model building. A strict correlation between the representations and their modular weights might be the right way to proceed. Useful shaping symmetries might be found within the traditional flavor symmetry instead.

The TD approach needs to make serious attempts for the construction of more explicit models. In particular, it would be useful to increase the number of explicit string constructions that ressemble the SM with gauge group SU(3) × SU(2) × U(1) and three families of quarks and leptons. This is important, as in generic string theory we might find huge classes of duality symmetries that might not survive in models with the properties of the SM. Of course, the size and nature of these large symmetry groups have to be explored. Modular invariance and its group SL(2,Z) are closely related to torus compactifications, which can be realized in orbifold compactifications and more generally in Calabi–Yau compactifications with elliptic fibrations. This can be described by the basic building blocks $T^{2}/Z_{K}$ with K=2,3,4,6, which have been studied previously [68]. Explicit string model building shows that such situations are possible, but require particular constellations for the Wilson lines needed for realistic model building. Such Wilson lines and other background fields might otherwise break modular symmetries in various ways [85,86,87]. In some orbifolds, only a subgroup of SL(2,Z) is unbroken, even without background fields [88], which opens up the possibility of finite modular flavor symmetries beyond $\Gamma_{K}^{\prime }$ [65,66,89]. However, a more general discussion has to go beyond SL(2,Z). A first step in the direction is the consideration of the Siegel modular group [78,90,91] or higher dimensional constructions [67,68,92,93]. Many exciting developments seem to be in front of us.

## Data Availability

No new data were created or analyzed in this study. Data sharing is not applicable to this article.

## Abbreviations

The following abbreviations are used in this manuscript:

TD	Top-Down
BU	Bottom-Up
SM	Standard Model
PMNS	Pontecorvo–Maki–Nakagawa–Sakata

## Appendix A. The Group *A*_4_ and its Double Cover *T*′

### Appendix A.1. A_4_

$A_{4}≅\left(Z_{2}\times Z_{2}\right)⋊Z_{3}$ (GAP Id [12,3]) is the alternating group of four elements and can also be understood as the rotational symmetry group of a regular tetrahedron. It contains 12 elements. $A_{4}$ has the irreducible representations $r\in \{1,1^{\prime },1^{\prime \prime },3\}$. With $\omega :=e^{2\pi i\;/\;3}$, its character table reads

class	$1C_{1}$	$3C_{2}$	$4C_{3}$	$4C_{3}^{\prime }$
representative	1	S	T	$T^{2}$
1	1	1	1	1
$1^{\prime }$	1	1	ω	$\omega^{2}$
$1^{\prime \prime }$	1	1	$\omega^{2}$	ω
3	3	−1	0	0

in terms of the generators S and T. They satisfy $S^{2}=T^{3}={\left(\mathrm{ST}\right)}^{3}=1$ and their representations $\rho_{r}$ can be expressed by

r	1	$1^{\prime }$	$1^{\prime \prime }$	3
$\rho_{r}\left(S\right)$	1	1	1	$\frac{1}{3}\left(\begin{array}{ccc} -1 & 2 & -2\\ 2 & -1 & -2\\ -2 & -2 & -1 \end{array}\right)$
$\rho_{r}\left(T\right)$	1	ω	$\omega^{2}$	$\mathrm{diag}(1,\omega ,\omega^{2})$

The $A_{4}$ product rules are

$$\begin{array}{cc} 1^{a}⊗1^{b} & =1^{c}\;\;\mathrm{with}\;\;c=a+b\;\;\;\;\mathrm{mod}\;3,\;\;1^{a}⊗3=3,\;\;3⊗3=1⊕1^{\prime }⊕1^{\prime \prime }⊕3⊕3, \end{array}$$

where a,b,c=0,1,2 correspond to the number of primes.

### Appendix A.2. T′

$T^{\prime }$ (GAP Id [24,3]) is the double cover of $A_{4}$ known also as the binary tetrahedral group. Its irreducible representations are $r\in \{1,1^{\prime },1^{\prime \prime },2,2^{\prime },2^{\prime \prime },3\}$. This group can be generated by two generators, S and T, satisfying $S^{4}=T^{3}={\left(\mathrm{ST}\right)}^{3}=1$ and $S^{2}T=TS^{2}$. This leads to the character table

class	$1C_{1}$	$1C_{2}$	$6C_{4}$	$4C_{3}$	$4C_{3}^{\prime }$	$4C_{6}$	$4C_{6}^{\prime }$
representative	1	$S^{2}$	S	T	$T^{2}$	$S^{2}T$	$S^{2}T^{2}$
1	1	1	1	1	1	1	1
$1^{\prime }$	1	1	1	ω	$\omega^{2}$	ω	$\omega^{2}$
$1^{\prime \prime }$	1	1	1	$\omega^{2}$	ω	$\omega^{2}$	ω
2	2	−2	0	−1	−1	1	1
$2^{\prime }$	2	−2	0	−ω	$-\omega^{2}$	ω	$\omega^{2}$
$2^{\prime \prime }$	2	−2	0	$-\omega^{2}$	−ω	$\omega^{2}$	ω
3	3	3	−1	0	0	0	0

Note that the triplet representation is unfaithful; it yields only $A_{4}≅T^{\prime }/Z_{2}$, where the normal $Z_{2}$ subgroup is generated by $S^{2}$. The representations can be expressed as

r	1	$1^{\prime }$	$1^{\prime \prime }$	2	$2^{\prime }$	$2^{\prime \prime }$	3
$\rho_{r}\left(S\right)$	1	1	1	$\Omega_{S}$	$\Omega_{S}$	$\Omega_{S}$	$\rho_{3}\left(S\right)$
$\rho_{r}\left(T\right)$	1	ω	$\omega^{2}$	${\left(\Omega_{T}{\tilde{\Omega }}_{T}\right)}^{*}$	$\Omega_{T}$	${\tilde{\Omega }}_{T}$	$\rho_{3}\left(T\right)$

where we defined the two-dimensional matrices

$$\Omega_{S}=-\frac{i}{\sqrt{3}}\left(\begin{array}{cc} 1 & \sqrt{2}\\ \sqrt{2} & -1 \end{array}\right),\;\Omega_{T}=\mathrm{diag}\left(1,\omega^{2}\right),\;{\tilde{\Omega }}_{T}=\mathrm{diag}\left(\omega ,1\right)$$

and the three-dimensional representation is given (as in $A_{4}$) by

$$\rho_{3}\left(S\right)=\frac{1}{3}\left(\begin{array}{ccc} -1 & 2 & -2\\ 2 & -1 & -2\\ -2 & -2 & -1 \end{array}\right)\;\mathrm{and}\;\rho_{3}\left(T\right)=\mathrm{diag}\left(1,\omega ,\omega^{2}\right)\;.$$

Finally, the tensor products of the $T^{\prime }$ irreducible representations are given by

$$\begin{array}{cc} 1^{a}⊗1^{b} & =1^{c},\;1^{a}⊗2^{b}=2^{c},\;2^{a}⊗2^{b}=1^{c}⊕3\;\mathrm{with}\;c=a+b\;\mathrm{mod}\;3,\\ 1^{a}⊗3 & =3,\;2^{a}⊗3=2⊕2^{\prime }⊕2^{\prime \prime }\;\mathrm{and}\;3⊗3=1⊕1^{\prime }⊕1^{\prime \prime }⊕3⊕3, \end{array}$$

where a,b,c=0,1,2 correspond to the number of primes. The Clebsch–Gordan coefficients can be found, e.g., in [94].

## Appendix B. Group Theory Elements of Larger Groups

### Appendix B.1. *Δ(54)*

$\Delta \left(54\right)≅\left(Z_{3}\times Z_{3}\right)⋊S_{3}≅\left(\left(Z_{3}\times Z_{3}\right)⋊Z_{3}\right)⋊Z_{2}$ (GAP Id [54,8]) has 54 elements, which can be generated by three generators, A,B,C, satisfying the presentation $A^{3}=B^{3}=C^{2}={\left(\mathrm{AB}\right)}^{3}={\left({\mathrm{AB}}^{2}\right)}^{3}={\left(\mathrm{AC}\right)}^{2}={\left(\mathrm{BC}\right)}^{2}=1$. Its irreducible representations are two singlets, four doublets, and two triplets, plus their complex conjugates. Together, they lead to the character table

class	$1C_{1}$	$9C_{2}$	$6C_{3}$	$6C_{3}^{\prime }$	$6C_{3}^{\prime \prime }$	$6C_{3}^{\prime \prime \prime }$	$1C_{3}$	$1C_{3}^{\prime }$	$9C_{6}$	$9C_{6}^{\prime }$
repr.	1	C	A	B	AB	${\mathrm{AB}}^{2}$	${\left(\mathrm{ABC}\right)}^{2}$	${\left(\mathrm{ACB}\right)}^{2}$	ABC	ACB
1	1	1	1	1	1	1	1	1	1	1
$1^{\prime }$	1	−1	1	1	1	1	1	1	−1	−1
$2_{1}$	2	0	2	−1	−1	−1	2	2	0	0
$2_{2}$	2	0	−1	2	−1	−1	2	2	0	0
$2_{3}$	2	0	−1	−1	−1	2	2	2	0	0
$2_{4}$	2	0	−1	−1	2	−1	2	2	0	0
$3_{1}$	3	1	0	0	0	0	3ω	$3\omega^{2}$	$\omega^{2}$	ω
$3_{2}$	3	−1	0	0	0	0	3ω	$3\omega^{2}$	$-\omega^{2}$	−ω
${\bar{3}}_{1}$	3	1	0	0	0	0	$3\omega^{2}$	3ω	ω	$\omega^{2}$
${\bar{3}}_{2}$	3	−1	0	0	0	0	$3\omega^{2}$	3ω	−ω	$-\omega^{2}$

The doublets are unfaithful representations, which yield the quotient group $S_{3}≅\Delta \left(54\right)/Z_{3}\times Z_{3}$, where the normal subgroup $Z_{3}$ × $Z_{3}$ can be generated by A and ${\mathrm{BAB}}^{2}A^{2}$. In the irreducible representations, the Δ(54) generators can be expressed as

r	1	$1^{\prime }$	$2_{1}$	$2_{2}$	$2_{3}$	$2_{4}$	$3_{1}$	$3_{2}$	${\bar{3}}_{1}$	${\bar{3}}_{2}$
$\rho_{r}\left(A\right)$	1	1	$1_{2}$	$\Omega_{2}$	$\Omega_{2}$	$\Omega_{2}$	ρ(A)	ρ(A)	ρ(A)	ρ(A)
$\rho_{r}\left(B\right)$	1	1	$\Omega_{2}$	$1_{2}$	$\Omega_{2}$	$\Omega_{2}^{*}$	ρ(B)	ρ(B)	$\rho {\left(B\right)}^{*}$	$\rho {\left(B\right)}^{*}$
$\rho_{r}\left(C\right)$	1	−1	$S_{2}$	$S_{2}$	$S_{2}$	$S_{2}$	ρ(C)	−ρ(C)	ρ(C)	−ρ(C)

where the doublet representations are generated by

$$1_{2}=\left(\begin{array}{cc} 1 & 0\\ 0 & 1 \end{array}\right),\;\Omega_{2}=\left(\begin{array}{cc} \omega^{2} & 0\\ 0 & \omega \end{array}\right),\;S_{2}=\left(\begin{array}{cc} 0 & 1\\ 1 & 0 \end{array}\right),$$

and the triplets by

$$\rho \left(A\right)=\left(\begin{array}{ccc} 0 & 1 & 0\\ 0 & 0 & 1\\ 1 & 0 & 0 \end{array}\right),\;\rho \left(B\right)=\left(\begin{array}{ccc} 1 & 0 & 0\\ 0 & \omega & 0\\ 0 & 0 & \omega^{2} \end{array}\right),\;\rho \left(C\right)=\left(\begin{array}{ccc} 1 & 0 & 0\\ 0 & 0 & 1\\ 0 & 1 & 0 \end{array}\right).$$

It is useful to list the nontrivial tensor products of Δ(54) irreducible representations:

$$\begin{array}{cc} 1^{\prime }⊗1^{\prime } & =1,\;\;1^{\prime }⊗2_{k}=2_{k},\;\;1^{\prime }⊗3_{1}=3_{2},\;\;1^{\prime }⊗3_{2}=3_{1},\;\;1^{\prime }⊗{\bar{3}}_{1}={\bar{3}}_{2},\;\;1^{\prime }⊗{\bar{3}}_{1}={\bar{3}}_{2},\\ 2_{k}⊗2_{k} & =1⊕1^{\prime }⊕2_{k},\;\;2_{k}⊗2_{\ell }=2_{m}⊕2_{n}\;\mathrm{with}\;k\neq \ell \neq m\neq n,\;k,\ell ,m,n=1,\ldots ,4,\\ 2_{k}⊗3_{\ell } & =3_{1}⊕3_{2},\;\;2_{k}⊗{\bar{3}}_{\ell }={\bar{3}}_{1}⊕{\bar{3}}_{2}\;\mathrm{for}\;\mathrm{all}\;\;k=1,\ldots ,4,\;\ell =1,2,\\ 3_{\ell }⊗3_{\ell } & ={\bar{3}}_{1}⊕{\bar{3}}_{1}⊕{\bar{3}}_{2},\;\;3_{1}⊗3_{2}={\bar{3}}_{2}⊕{\bar{3}}_{2}⊕{\bar{3}}_{1},\;\;3_{1}⊗{\bar{3}}_{1}=1⊕2_{1}⊕2_{2}⊕2_{3}⊕2_{4},\\ 3_{1}⊗{\bar{3}}_{2} & =1^{\prime }⊕2_{1}⊕2_{2}⊕2_{3}⊕2_{4},\;\;3_{2}⊗{\bar{3}}_{1}=1^{\prime }⊕2_{1}⊕2_{2}⊕2_{3}⊕2_{4},\\ 3_{2}⊗{\bar{3}}_{2} & =1⊕2_{1}⊕2_{2}⊕2_{3}⊕2_{4},\;\;{\bar{3}}_{\ell }⊗{\bar{3}}_{\ell }=3_{1}⊕3_{1}⊕3_{2},\;\;{\bar{3}}_{1}⊗{\bar{3}}_{2}=3_{2}⊕3_{2}⊕3_{1}\;. \end{array}$$

The explicit Clebsch–Gordan coefficients can be found, e.g., in [94].

### Appendix B.2. *Δ(27)*

The group $\Delta \left(27\right)≅\left(Z_{3}\times Z_{3}\right)⋊Z_{3}$ (GAP Id [27,3]) can be obtained from Δ(54), excluding the $Z_{2}$ generator C. Hence, the generators A and B yielding the 27 elements of the group are constrained to fulfill only the subset of conditions $A^{3}=B^{3}={\left(\mathrm{AB}\right)}^{3}={\left({\mathrm{AB}}^{2}\right)}^{3}=1$. By excluding C in the Δ(54) character table, we observe that the Δ(27) representations arise from the trivial singlet, the doublets, and combinations of triplets of Δ(54). One can show that they break into nine singlets and two triplets, which describe the character table

class	$1C_{1}$	$3C_{3}^{a}$	$3C_{3}^{b}$	$3C_{3}^{c}$	$3C_{3}^{d}$	$3C_{3}^{e}$	$3C_{3}^{f}$	$3C_{3}^{g}$	$3C_{3}^{h}$	$3C_{3}^{i}$	$1C_{3}$
repr.	1	A	B	$A^{2}$	$B^{2}$	AB	${\mathrm{AB}}^{2}$	${\left(\mathrm{AB}\right)}^{2}$	${\mathrm{BA}}^{2}$	$A^{2}B^{2}\mathrm{AB}$	$A{\left(\mathrm{AB}\right)}^{2}B$
$1_{0,0}$	1	1	1	1	1	1	1	1	1	1	1
$1_{0,1}$	1	1	ω	1	$\omega^{2}$	ω	$\omega^{2}$	$\omega^{2}$	ω	1	1
$1_{0,2}$	1	1	$\omega^{2}$	1	ω	$\omega^{2}$	ω	ω	$\omega^{2}$	1	1
$1_{1,0}$	1	ω	1	$\omega^{2}$	1	ω	ω	$\omega^{2}$	$\omega^{2}$	1	1
$1_{1,1}$	1	ω	ω	$\omega^{2}$	$\omega^{2}$	$\omega^{2}$	1	ω	1	1	1
$1_{1,2}$	1	ω	$\omega^{2}$	$\omega^{2}$	ω	1	$\omega^{2}$	1	ω	1	1
$1_{2,0}$	1	$\omega^{2}$	1	ω	1	$\omega^{2}$	$\omega^{2}$	ω	ω	1	1
$1_{2,1}$	1	$\omega^{2}$	$\omega^{2}$	ω	ω	ω	1	$\omega^{2}$	1	1	1
$1_{2,2}$	1	$\omega^{2}$	ω	ω	$\omega^{2}$	1	ω	1	$\omega^{2}$	1	1
3	3	0	0	0	0	0	0	0	0	3ω	$3\omega^{2}$
$\bar{3}$	3	0	0	0	0	0	0	0	0	$3\omega^{2}$	3ω

Here we immediately see that the singlets $1_{r,s}$, r,s=0,1,2, have the representations $\rho_{r,s}\left(A\right)=\omega^{r}$ and $\rho_{r,s}\left(B\right)=\omega^{s}$. Further, the triplet representations are given by $\rho_{3}\left(A\right)=\rho_{\bar{3}}\left(A\right)=\rho \left(A\right)$ and $\rho_{3}\left(B\right)=\rho_{\bar{3}}{\left(B\right)}^{*}=\rho \left(B\right)$, in terms of the Δ(54) matrices.

Finally, the tensor products of Δ(27) irreducible representations are given by

$$\begin{array}{cc} 1_{r,s}⊗1_{r^{\prime },s^{\prime }} & =1_{r^{\prime \prime },s^{\prime \prime }}\;\mathrm{with}\;r^{\prime \prime }=r+r^{\prime }\;\;\;\mathrm{mod}\;3,\;s^{\prime \prime }=s+s^{\prime }\;\;\;\mathrm{mod}\;3,\\ 1_{r,s}⊗3 & =3,\;\;1_{r,s}⊗\bar{3}=\bar{3}\;\mathrm{for}\;\mathrm{all}\;\;r,s=0,1,2,\\ 3⊗3 & =\bar{3}⊕\bar{3}⊕\bar{3},\;\;\bar{3}⊗\bar{3}=3⊕3⊕3\;\mathrm{and}\;3⊗\bar{3}=\sum_{r,s}1_{r,s}. \end{array}$$

## Appendix C. *T*′ Modular Forms

The vector space of SL(2,Z) modular forms of weight 1 associated with $T^{\prime }≅\Gamma_{3}^{\prime }=\mathrm{SL}\left(2,Z\right)/\Gamma \left(3\right)$ can be spanned by [63]

$${\hat{e}}_{1}\left(M\right)\;:=\;\frac{\eta^{3}\left(3\;M\right)}{\eta (M)}\;\mathrm{and}\;{\hat{e}}_{2}\left(M\right)\;:=\;\frac{\eta^{3}\left(M/3\right)}{\eta (M)}\;,$$

in terms of the Dedekind η-function of the modulus *M*. One can show that the combinations

$$Y^{\left(1\right)}\left(M\right)\;=\;\left(\begin{array}{c} {\hat{Y}}_{1}\left(M\right)\\ {\hat{Y}}_{2}\left(M\right) \end{array}\right)\;:=\;\left(\begin{array}{cc} -3\sqrt{2} & 0\\ 3 & 1 \end{array}\right)\left(\begin{array}{c} {\hat{e}}_{1}\left(M\right)\\ {\hat{e}}_{2}\left(M\right) \end{array}\right)$$

transform under γ∈SL(2,Z) as

$$Y^{\left(1\right)}\left(M\right)\;\overset{\gamma }{⟶}\;\left(cM+d\right)\;\rho_{2^{\prime \prime }}\left(\gamma \right)\;Y^{\left(1\right)}\left(M\right)\;,$$

i.e., building a $2^{\prime \prime }$ representation $\rho_{2^{\prime \prime }}$ of $\Gamma_{3}^{\prime }≅T^{\prime }$, which is given in Appendix A.2. Higher-weight modular forms of $T^{\prime }$ are derived from $Y^{\left(1\right)}\left(M\right)$ by the products of this basic vector-valued modular form, such that $Y^{\left(n+m\right)}\left(M\right)=Y^{\left(n\right)}\left(M\right)⊗Y^{\left(m\right)}\left(M\right)$. For instance, the modular forms of weight 2 are obtained from $Y^{\left(2\right)}\left(M\right)=Y^{\left(1\right)}\left(M\right)⊗Y^{\left(1\right)}\left(M\right)$, which build the $T^{\prime }$ (and $A_{4}$) triplet ${\left({\hat{Y}}_{2}{\left(M\right)}^{2},\sqrt{2}{\hat{Y}}_{1}\left(M\right){\hat{Y}}_{2}\left(M\right),{\hat{Y}}_{1}{\left(M\right)}^{2}\right)}^{T}=:{\left({\hat{X}}_{1},{\hat{X}}_{2},{\hat{X}}_{3}\right)}^{T}$ (other choices for the $T^{\prime }$ Clebsch–Gordan coefficients lead to different but unimportant signs). The expected singlet from $2^{\prime \prime }⊗2^{\prime \prime }=1^{\prime }⊕3$ vanishes, and we observe the known relation ${\hat{X}}_{2}^{2}-2{\hat{X}}_{1}{\hat{X}}_{3}=0$, which can lead to interesting consequences [95].
